# Home care nurses more positive about the palliative care that is provided and their own competence than hospital nurses: a nationwide survey

**DOI:** 10.1186/s12904-021-00866-4

**Published:** 2021-10-28

**Authors:** Chantal Y. Joren, Anke J.E. de Veer, Kim de Groot, Anneke L. Francke

**Affiliations:** 1 Netherlands Institute for Health Services Research (Nivel), PO Box 1568, Utrecht, 3513 CR The Netherlands; 2 Thebe Wijkverpleging (Home care organisation), Lage Witsiebaan 2a, Tilburg, 5042 DA The Netherlands; 3 Department of Public and Occupational Health, Amsterdam Public Health Research Institute (APH), Amsterdam UMC, Vrije Universiteit Amsterdam, Van der Boechorststraat 7, Amsterdam, 1081 BT The Netherlands; 4 Expertise Center for Palliative Care Amsterdam, Amsterdam UMC, VU Medical Center, Amsterdam, Netherlands

**Keywords:** Nurses, Palliative care, Home care, Hospitals, Competencies, Quality of care

## Abstract

**Background:**

People often prefer to stay at home until the end of life, but hospital admissions are quite common. In previous research bereaved relatives were found to be less positive about palliative care in hospital. However, it was not known how the content and quality of palliative care differ between home care and hospitals from the perspectives of hospital nurses and home care nurses and how palliative care in these settings could be improved.

**Methods:**

A survey was held among hospital and home care nurses, recruited from a nationwide Nursing Staff Panel and through open calls on social media and in an online newsletter. The pre-structured online survey included questions on the palliative care provided, the quality of this care and the respondent’s perceived competence in providing palliative care. The questionnaire was completed by 229 home care nurses and 106 hospital nurses.

**Results:**

Most nurses provided palliative care in the physical and psychological domains, fewer provided care in the social and spiritual domains. A higher percentage of home care nurses stated that they provided care in these domains than hospital nurses. Overall, 70% of the nurses rated the quality of palliative care as very good to excellent. This percentage was higher among home care nurses (76.4%) than hospital nurses (59.4%). Moreover, a higher percentage of home care nurses (94.4%) stated they felt competent to a great extent to provide palliative care compared to hospital nurses (84.7%). Competencies regarding the physical domain were perceived as better compared to the competencies concerning the other domains. The nurses recommended paying more attention to inter-professional collaboration and communication, timely identification of the palliative phase and advance care planning, and more time available for palliative care patients.

**Conclusion:**

Although the quality of palliative care was rated as very good to excellent by nurses, improvements can still be made, particularly regarding palliative care in hospitals. Although patients often prefer to die at home rather than in hospital, still a considerable number of people do die in hospital; therefore hospital nurses must also be trained and be able to provide high-quality palliative care.

## Background

High-quality palliative care should be accessible in all care settings [1, 2], and should be provided in the setting preferred by the patient, if feasible [2]. People often prefer to stay at home until the end of their lives [1, 3–5]. In addition, international health policy documents, e.g. from the World Health Organisation, advocate providing palliative care in primary care as much as possible [6]. Dutch healthcare policy also assumes that patients should be able to stay at home as long as possible [7]; moreover, its guiding principle is that palliative care should be an integral part of generalist care, e.g. provided by home care nurses and general practitioners. Dutch home care nurses provide nursing care, including palliative care, for patients living independently in the community. If needed, home care nurses and general practitioners can consult specialised palliative care teams [8].

However, remaining at home until death is not always realized. In European countries and Israel in the period 2006 to 2013, 22-49% of patients died at home, while 31-63% died in hospital [9]. In the Netherlands in 2017, 36% of patients with potential palliative care needs died at home and 20% in hospital [10]. If patients are admitted to a hospital, then the hospital nurses must also be able to provide good palliative care in cooperation with medical specialists. However, previous survey research indicated that bereaved relatives of patients who died in a Dutch hospital were less positive about the quality of palliative care than relatives of patients who died at home [11]. In other countries too – namely England, Canada and Japan - higher percentages of bereaved relatives appraised the care at the end of life at home positively compared with bereaved relatives whose loved one died in a hospital [12–14]. The differences in perceived quality of care raise questions about possible differences between the palliative care provision at home and in a hospital and whether *nurses* also perceive a difference in quality of care. In addition, it is important to investigate the extent to which nurses feel competent in providing palliative care, as this could influence both the quality and the nature of the care provided. To date it was not known whether nurses in hospitals also differ from nurses in home care in their experiences and perspectives regarding palliative care. This article therefore answers the following research questions:

1. What palliative care is provided by nurses in home care and in hospitals?
2. How do these nurses rate the quality of the palliative care that is provided and how can this quality be improved according to the nurses?
3. To what extent do nurses in home care and hospitals feel competent in providing palliative care?

## Methods

### Design

A cross-sectional survey study was conducted. The online survey was held in July 2020 among nurses working in a hospital or home care.

### Recruitment and sample

The first method used for recruitment was to approach the participants of a pre-existing nationwide research panel, the Nursing Staff Panel [15]. The Nursing Staff Panel consists of nurses working in various healthcare sectors, but for this study we only selected those working in a hospital or in home care. Recruitment for membership of the Panel was mainly through a random sample of Dutch healthcare employees that was provided by two pension funds [15]. All Panel members consent to participate regularly in survey research addressing issues relating to their work. For this study, an email with a link to the survey was sent to all Nursing Staff Panel members working in home care (*n*=468) or working in hospitals (*n*=430). At one and two weeks after the initial email was sent, an email reminder was sent to non-respondents to improve response rates.

A second recruitment method was to use social media and an online newsletter. An open call to participate in the survey together with a link to the survey was sent out via Twitter, LinkedIn, Facebook groups for nurses and an online newsletter of IKNL - the Netherlands comprehensive cancer organisation.

On starting the online survey, potential participants were asked if they had been involved in palliative care in the past two years. If they had been, they were invited to complete the whole questionnaire and were included in the study. If they had not been involved in palliative care, the questionnaire automatically ended and they were excluded from the study.

### Questionnaire

The pre-structured online survey included questions on the characteristics of the nurses, the palliative care provided, the quality of the care provided and the respondent’s own perceived competence in providing palliative care. The questionnaire started with the statement that the palliative phase usually starts before the terminal phase.

The palliative care provided was measured by 23 items, each describing an aspect of palliative care. The aspects were derived from the Netherlands Quality Framework for Palliative care [16], a national guide for palliative care at home [17], and a questionnaire previously used in the Nursing Staff Panel about palliative care [18, 19]. The items concerned aspects of care for the patients, support for the relatives and aftercare, and were related to the physical, psychological, social and spiritual care domains. Nurses could indicate if they provided the care aspects described for the majority of their palliative care patients (yes or no). The care aspects addressed in the questionnaire are listed in Tables 2, 3 and 4 and are divided into four, sometimes overlapping, palliative-care domains: physical, psychological, social and spiritual care. These domains cover the following kinds of care:

- Physical care: providing physical care and managing physical symptoms. Technical nursing care is a subset of this domain.
- Psychological care: dealing with psychological complaints (such as sadness and anxiety) and giving emotional support.
- Social care: paying attention to the social functioning of the patient and their relatives (for example regarding changing roles, communication with family and friends, or financial concerns).
- Spiritual care: paying attention to the spiritual requirements of the patient and their family (regarding religious, meaningful and existential questions).

In addition, the survey questionnaire addressed the perceived quality of palliative care that patients received in general in the nurses’ team or in their organisation. The quality of palliative care was rated on a five-point scale (1=poor to 5=excellent). If the score was less than 4 (very good), nurses could indicate in an open question how the quality of palliative care could be improved. The perceived competence in providing palliative care was first measured overall and then individually for the four palliative care domains: physical, psychological, social and spiritual care. There was a short explanation for each domain of what that care domain covers. The questions on competence were measured on a four-point scale (1=not at all, to 4=to a great extent).

### Analysis

We used descriptive statistics to describe the characteristics of the respondents, the palliative care provided, the perceived quality of care and the degree of competence. In addition, Chi-square tests or Fisher Exact tests (in case of limited number of cases) were used to analyse differences between nurses in home care and nurses in hospitals. For both the Chi-square and Fisher Exact tests, a p-value of 0.05 was used to indicate statistical significance. The data was analysed using STATA version 15.0.

The answers on the open question on how the palliative care could be improved were analysed using a qualitative descriptive analysis, whereby the aspects in which nurses considered improvements to be necessary were derived directly from the topics mentioned in the answers of the respondents.

## Results

A total of 273 nurses from the Nursing Staff Panel (response rate 30.4%) completed the questionnaire and 62 nurses completed the questionnaire via the open link posted on social media or in the online newsletter. As shown in Table 1, the majority of the respondent nurses were female and the mean age was 48.6. Most of the nurses worked at least 16 hours a week and had a bachelor’s degree. Nurses in hospitals worked more hours a week compared to nurses in home care (*p*<0.05). Home care nurses and hospital nurses did not differ significantly in terms of gender, age or educational level (*p*>0.05).

**Table 1:** Characteristics of the respondents (*n*=335)

Variable	Home care ***n***= 229 (n, %)	Hospital ***n***=106 (n, %)
**Gender**		
Female	215 (93.9)	94 (88.7)
Male	14 (6.1)	12 (11.3)
**Age (years)**	Mean: 49.6	Mean:46.6
<=35	43 (18.8)	26 (24.5)
36-45	26 (11.4)	21 (19.8)
46-55	69 (30.1)	27 (25.5)
>=56	91 (39.7)	32 (30.2)
**Number of hours employed**	Mean: 26.2	Mean: 29.4
<=16 hours	21 (10.0)	5 (5.3)
>16 & <32 hours	130 (61.9)	39 (41.1)
>=32 hours	59(28.1)	51 (53.7)
**Level of education**		
Registered nurse, secondary vocational qualification (EQF^a^=4)	87 (38.2)	42 (41.2)
Registered nurse, bachelor’s degree (EQF^a^ =6)	141 (61.8)	60 (58.8)

^a^ European Qualifications Framework

### Palliative care provided to patients

The vast majority of respondent nurses generally provided care in the physical and psychological domains of palliative care (Table 2). In the physical care domain, nurses mostly provided technical nursing care (89.2%) and physical care in the sense of helping with washing, eating or dressing (83.5%). Fewer nurses provided social and spiritual care. Half of the nurses held discussions with the patient about the patient’s changing roles and responsibilities in their family. Fewer nurses (28.7%) provided support in arranging practical matters regarding the death.

**Table 2:** Percentage of nurses that provided specific aspects of palliative care to majority of their patients

With the majority I have...	Hospital (***n***=105)	Home care (***n***=229)	Total (***n***=334)	P*
**Domain: physical care**				
- Provided technical care (e.g. catheter or stoma care, wound care, administration of medication)	77.1	94.8	89.2	0.000
- Provided physical care (e.g. help with washing, eating, dressing)	70.5	89.5	83.5	0.000
- Provided complementary care (e.g. massage, heat application)	11.4	16.6	15.0	0.219
- Talked with the patient about the physical problems and required care	62.9	91.3	82.3	0.000
- Discussed end-of-life decisions (e.g. not initiating or discontinuing treatment, no more hospital admissions, euthanasia, assisted suicide) with the patient	55.2	82.1	73.7	0.000
**Domain: psychological care**				
- Paid attention to the patient’s psychological and emotional wellbeing (such as anxiety, sadness)	81.9	98.3	93.1	0.000
- Supported the patient in dealing with the impending death, saying goodbye to family, etc.	65.7	91.3	83.2	0.000
**Domain: social care**				
- Discussed with the patient the changing roles and responsibilities in the family, communication with family and friends, financial concerns	31.4	58.5	50.0	0.000
- Provided the patient with support in arranging practical matters regarding death (e.g. a memorial service, clothing that the client wants to wear after the death)	12.4	36.2	28.7	0.000
**Domain: spiritual care**				
- Talked to the patient about existential questions e.g. “what is still important to me in life?”, “why has this happened to me?”	45.7	68.6	61.4	0.000
- Offered the patient support in the practice of their religion or beliefs	33.3	45.4	41.6	0.038

* Chi-square test

As shown in Table 2, a higher percentage of home care nurses stated that they provided care in the physical and psychological domains than hospital nurses (*p*<0.05). Almost all home care nurses provided technical nursing care and physical care (respectively 94.8 and 89.5%), while 77.1% of the hospital nurses reported that they provided technical care and 70.5% physical care.

Also, a higher percentage of home care nurses discussed end-of-life decisions with patients, such as about not initiating or discontinuing treatment or no more hospital admissions. Although a smaller percentage of nurses in both home care and hospitals provided care in the social and spiritual care domains, relatively more nurses in home care provided care in these care domains than hospital nurses. Relatively more home care nurses discussed the changing roles and responsibilities within the family with patients, e.g., regarding communication with family and friends and financial concerns. Also, more home care nurses than hospital nurses talked to patients about existential questions, such as “What is still important to me in life?” or “Why has this happened to me?”

### Support for relatives

In general, most nurses stated they supported the relatives of palliative-care patients in the physical and psychological care domains (Table 3). Almost all of the nurses (91.6%) supported relatives in dealing with the impending death of the patient. Most (81.4%) spoke with relatives about the physical problems and the care required by the patient. Also, most nurses (77.3%) discussed end-of-life decisions, such as not initiating or discontinuing life-prolonging treatments. A smaller percentage stated that they supported relatives in the social and spiritual care domains. One third (34.1%) reported that they provided support in arranging practical matters relating to the death and 29.3% reported that they offered support to relatives in the practice of their religion.

**Table 3:** Percentages of nurses who reported that they provided support to relatives of palliative care patients

With the majority I have...	Hospital (***n***=105)	Home care (***n***=229)	Total (***n***=334)	P*t
**Domain: physical care**				
- Given the relatives support with the physical care of the patient	33.3	81.2	66.2	0.000
- Talked to the relatives about the physical problems and care required	62.9	90.0	81.4	0.000
- Discussed end-of-life decisions (e.g. not initiating or discontinuing treatment, no more hospital admissions, euthanasia, assisted suicide) with the relatives	64.8	83.0	77.3	0.000
**Domain: psychological care**				
- Supported the relatives in dealing with the impending death, saying goodbye to the patient, etc.	83.8	95.2	91.6	0.000
- Discussed the capacity, burden, values, wishes and needs of the informal caregiver in their role as a family member and offered support to prevent or reduce a possible overload	59.1	94.8	83.5	0.000
**Domain: social care**				
- Talked with the relatives about the changing roles and responsibilities in the family, communication with family and friends, financial concerns	31.4	60.3	51.2	0.000
- Provided support to relatives in arranging practical matters relating to death (for example, concerning a memorial service)	24.8	38.4	34.1	0.014
**Domain: spiritual care**				
- Talked to the relatives about existential questions (e.g. “why has this happened to me?”, “what can this experience mean for me?”	41.0	56.8	51.8	0.007
- Offered support to relatives in the practice of their religion or beliefs	27.6	30.1	29.3	0.640

* Chi-square test

Table 3 shows differences between nurses in home care and hospitals (*p*<0.05). A considerably larger percentage of home care nurses (81.2%) gave relatives support with the physical care of the patient compared to nurses in hospitals (33.3%). Also, a larger percentage of home care nurses (94.8%) than hospital nurses (59.1%) reported that they discussed the capacity, burden, values, wishes and needs of the family caregiver and offered support to prevent or reduce a possible overload. Moreover, more home care nurses reported that they supported relatives in the social and spiritual care domains than hospital nurses.

### Care provided after the patient’s death

Table 4 shows that about 40 per cent of the total group of nurses reported that they administered physical care to the deceased patient. In addition, almost half of the total group (49.0%) reported that they discussed with relatives what they needed to be able to get through the period of mourning and process the loss properly. About one fifth of nurses (21.0%) reported that they did not provide care for relatives in the period after the passing of the patient.

**Table 4:** Percentages of nurses who reported that they provided care after the death of the patient

With the majority I have…	Hospital (***n***=105)	Home care (***n***=228)	Total (***n***=333)	P*
- Administered physical care to the deceased patient (possibly together with the next of kin)	50.5	35.5	40.2	0.010
- Discussed with relatives what they need to be able to get through the period of mourning and process the loss properly	28.6	58.3	49.0	0.000
- Offered the relatives support in practical matters (for example concerning a memorial service)	22.9	17.5	19.2	0.253
- No care was given to relatives in the period after the death	22.9	20.2	21.0	0.577

* Chi-square test

Differences are found between nurses in home care and nurses in hospitals (*p*<0.05). Nurses in hospitals indicated more often that they administered physical care to the deceased patient compared with nurses in home care. However, nurses in home care were more likely to discuss with relatives what they needed to get through the period of mourning and process the loss properly.

### Quality of the palliative care provided

**Fig. 1:**
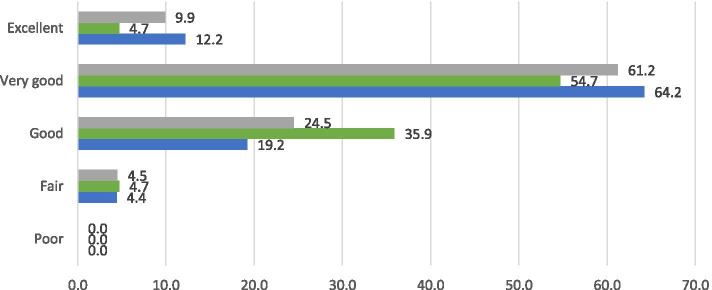
Quality of palliative care as rated by nurses, grey: total group (*n*=335), green: hospital (*n*=106, blue: home care (*n*=229) (%, Fisher Exact test *p*=0.004)

Overall, 70.1% of nurses scored the quality of palliative care within their team or organisation as very good to excellent (Fig. 1). No nurses rated the quality of palliative care as poor.

Differences are found between nurses in home care and nurses in hospitals (*p*<0.05). Of the nurses in home care, 76.4% scored the quality as very good to excellent, compared to 59.4% of hospital nurses.

Nurses who rated the palliative care as good or fair (29.0%) were asked to indicate what could be done to improve the quality of palliative care. Four main areas of improvement were revealed by the data.

#### Collaboration and communication

Nurses in both home care and hospitals mentioned that better peer and multidisciplinary collaboration and better communication could improve the quality of palliative care. As regards multidisciplinary collaboration, nurses mentioned better collaboration between the palliative-care team of hospitals, specialists, general practitioners and nurses. They also indicated that the communication has to improve between the healthcare professionals involved.

*“Better collaboration with different disciplines.” – Nurse in home care*

*“Communication between the healthcare providers currently involved and the healthcare providers to be involved.” – Nurse in hospital*

Nurses working in home care also mentioned better coordination between primary care and specialist care, more direct lines of communication with general practitioners and planning ahead for the weekend.

*“A little more anticipation of what can be done on weekends when the patient’s own general practitioner is not available, for everyone to be more alert to this.” – Nurse in home care*

#### Recognition and identification of the palliative phase

Both nurses working in home care and those working in hospitals mentioned that it is important to recognise and acknowledge the palliative phase in good time and to improve marking of the palliative phase. In addition, they said that many people see the dying phase and the palliative phase as the same thing.

*“Identification of the palliative phase can certainly still improve.” – Nurse in hospital*

*“Recognise and acknowledge the palliative phase in good time, give advanced care planning more attention, more multidisciplinary consultation and collaboration, especially between the general practitioner and home care nurses.” – Nurse in home care.*

#### Advance care planning

Nurses in both care settings mentioned that advance care planning often still does not take place. They indicated that advance care planning should be prioritised more often and should get more attention. Furthermore, they mentioned that the patient’s wishes must be clearly recorded. Nurses in hospitals also indicated that advance care planning should be applied earlier in outpatient care and clinical settings.

*“In general, the various phases are determined (too) late and there is still insufficient discussion of advanced care planning.” – Nurse in home care*

#### More time

Nurses in both home care and hospitals mentioned that more time would help improve the quality of palliative care. More time was needed to give patients sufficient attention and to be able to discuss things with patients and relatives. Nurses in home care indicated that they experience time pressure due to staff shortages and a high demand for care. In addition, they would like to be able to be paid for aftercare so that they could provide care after the patient has died.

*“Being able to charge for care hours after death so that aftercare can be provided.” – Nurse in home care*

Nurses in hospitals indicated that there is often not enough time or space to have proper conversations with patients. Moreover, they mentioned that they need more time to really give attention to patients, but that this is not always possible due to the care other patients need.

*“More time available per person would improve care. Now it has to be provided between care for other patients.” – Nurse in hospital*

### Degree to which nurses feel competent

**Fig. 2:**
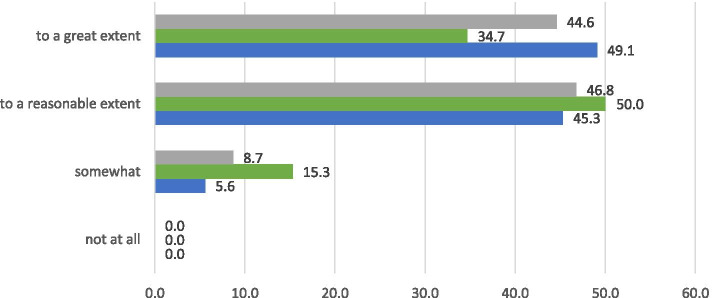
Overall perceived competence, grey: total group (*n*=335), green: hospital (*n*=106, blue: home care (*n*=229) (%, Fisher Exact test *p*=0.006)

Figure 2 indicates that almost all nurses (91.4%) felt competent to a reasonable or great extent to provide palliative care. None of the nurses said that they did not feel at all competent.

More nurses in home care (94.4%) indicated that they felt competent to a reasonable or great extent to provide palliative care, compared to nurses in hospitals (84.7%) (*p*<0.05).

According to the data in Table 5, almost three quarters of the nurses (71.5%) felt competent to a great extent to provide palliative care in the physical care domain. A smaller proportion of nurses felt competent to a great extent to provide care in the psychological, social and spiritual care domains.

**Table 5:** Perceived competence by nurses per palliative care domain (%)

When I provide palliative care, I feel competent in…	Hospital (***n***=97)	Home care (***n***=212-214)	Total (***n***=309-311)	P*
**Domain: physical care** Providing physical care and managing common physical symptoms				0.000
- Not at all	6.2	-	1.9	
- Somewhat	3.1	0.5	1.3	
- To a reasonable extent	26.8	24.5	25.2	
- To a great extent	63.9	75.0	71.5	
**Domain: psychological care** Dealing with psychological complaints (such as sadness and anxiety)				0.296
- Not at all	1.0	0.5	0.7	
- Somewhat	15.5	9.9	11.6	
- To a reasonable extent	53.6	52.1	52.6	
- To a great extent	29.9	37.6	35.2	
**Domain: social care** Paying attention to the social functioning of the patient and their relatives (such as changing roles, communication with family and friends, any financial concerns, wishes and goals still to be achieved)				0.000
- Not at all	3.1	0.9	1.6	
- Somewhat	27.8	9.9	15.5	
- To a reasonable extent	44.3	47.4	46.5	
- To a great extent	24.7	41.8	36.5	
**Domain: spiritual care** Giving attention to spirituality of the patient and their family (religious, meaningful and existential questions)				0.004
- Not at all	8.3	2.3	4.2	
- Somewhat	25.8	14.0	17.7	
- To a reasonable extent	41.2	54.2	50.2	
- To a great extent	24.7	29.4	28.0	

*Fisher Exact test

Nurses in home care were more likely to feel competent to a great extent in providing care in the physical, social and spiritual care domains compared to nurses in hospitals (*p*<0.05).

## Discussion

In this study we included home care nurses and hospital nurses who had been involved in palliative care in the past two years. Our study showed that overall, the care these nurses provided mostly concerned the physical and psychological domains of palliative care. Fewer nurses reported that they provided care in the social and spiritual domains.

Relatively more nurses in home care reported that they provided their patients with physical, psychological, social and spiritual care at the end of life compared to nurses in hospitals. An explanation for this difference could be that nurses working in hospitals have easier access to other multidisciplinary team members, such as a chaplain or psychologist, compared to nurses working in home care. This could have influenced the type of care provided by the nurses. A second explanation for this difference between nurses in home care and hospitals could be that in hospitals nurses identify that a patient is in need of palliative care at a later point in the illness trajectory. Bergenholtz et al. found in their study that in general a palliative-care approach in hospitals was only observed in patients whose death was imminent [20]. Gott et al. also found that transition to a palliative care approach in hospitals typically occurred close to death [21]. In line with these studies, hospital nurses in our study indicated that the identification that the patient is in the palliative phase, and subsequently advance care planning, could still improve. A third explanation for the differences in the spiritual and psychological care being provided could be the difference in the relationship between nurse and patient: when a patient is cared for in the patient’s own home the nurse is the guest, whereas in the hospital the patient is the guest. For this reason, it is plausible that patients at home feel more at ease and able to discuss existential questions or speak about their impending death, and that this influences the provision of spiritual and psychological care.

Relatively more nurses in hospitals reported that they administered physical care to the deceased patient, compared to nurses in home care. A plausible explanation for this result can be differences in procedures. When someone dies at home, an undertaker often takes care of the body, whereas that is a task for nurses in a hospital. Also, nurses in home care are not always present when the patient passes away, whereas in hospitals nurses are always present or nearby when this happens.

We also showed that large majorities of nurses in both home care and in hospitals provided technical-physical care for patients in need of palliative care, such as catheter or stoma care, wound care or the administration of medication. However, a higher percentage of home care nurses provided technical care (94.8%) compared to nurses in hospitals (77.1%). This finding was unexpected, since previous research indicated that in general patients who are admitted to hospital at the end of life experience more symptoms compared to patients at home [22, 23], and therefore we presumed that hospital nurses would be more likely to deliver technical care. A possible partial explanation for this unexpected finding could be that there has been a shift from hospital care to home care in the main groups of palliative-care patients in recent years [24, 25], although this is not reflected in a substantial increase in the percentages of patients dying at home. Therefore, it is plausible that technical-physical care has also gained a larger role in home care.

Our study also indicated differences between home care nurses and hospital nurses regarding the perceived quality of palliative care. A considerably larger percentage of nurses in home care rated the palliative care provided in their team or organisation as very good to excellent, compared to nurses in hospital (respectively 76.4 and 59.4%). This finding is in line with studies from the perspective of bereaved relatives in various countries [11–14]; this includes a study performed in the Netherlands, which showed that relatives of patients who died in hospitals were less positive about the quality of palliative care than relatives of patients who died at home [11].

Various studies gave indications as to why hospital professionals would not be best suited to provide high-quality palliative care. For instance, Gardiner et al. showed that inadequate staffing levels and time pressures affect the ability of hospital staff to provide good palliative care [26]. Lack of time was also described by nurses in our study, e.g. not having enough time for proper conversations with patients. However, in our study both hospital nurses and home care nurses said that they needed more time to provide good palliative care. Time restrictions were related to staff shortages and a high demand for care. Previous research from Sekse et al. also concluded that in both hospitals and home care there is a lack of time, which has an adverse effect on the quality of palliative care [27]. Hence the fact that hospital nurses were less positive than home care nurses about the quality of palliative care cannot be fully explained by the limited time available.

In addition, we found that more nurses felt competent in the physical care domain than in the psychological, social and spiritual domains of palliative care. This is in line with previous research by Sawatzky et al., who also found nurses felt relatively less competent in addressing psychological, social and spiritual needs [28]. Nevertheless, it is known that at the end of life, patients are often in need of care in the psychological, psychosocial and spiritual care domains as well as the physical domain [29]. Those four care domains are listed in the World Health Organisation’s description of palliative care and in the definition of palliative care in the Netherlands Quality Framework for Palliative care [16, 30]. However, our study not only indicated that nurses felt less competent in the psychological, social and spiritual domains, but also suggested that nurses provided relatively little social and spiritual care for patients at the end of life. Nurses in hospitals in particular felt less competent and provided less social and spiritual care. Considering that all four care domains (physical, psychological, social and spiritual) have been linked to the quality of life [31], this could have influenced the assessments of the quality of palliative care.

### Strengths and limitations

A methodological strength of this study is the recruitment of respondents through the nationally representative Nursing Staff Panel, in addition to open calls on social media. As a result we had a nationwide study sample. A second strength is the addition of an open question about the improvement of the quality of palliative care, which gave further insights into the quantitative results.

A limitation of this study is that we do not know if there are differences between the patient groups who received palliative care in home care and those who received palliative care in hospitals. This could have influenced the palliative care provided by nurses and the rated quality of care.

Also, the response rate is relatively low. This can partly be explained by the inclusion criteria, as only nurses who had provided palliative care in the past two years were asked to fill in the questionnaire. However, as the survey took place during the Covid-19 pandemic, this could also have negatively influenced the response rate.

### Implications

Although the nursing staff were positive about the palliative care provided, still improvements could be made, in particular regarding palliative care in hospitals. We recommend paying extra attention to education and training in the psychological, social and spiritual care domains for nurses who provide palliative care. Based on the reported areas for improvement, we also recommend that both nurses in home care and hospitals and care organisations invest more in collaboration and communication between nurses and physicians, the timely identification of the palliative phase and advance care planning, and in arranging enough time for nurses to provide high-quality care.

## Conclusion

Home care nurses are more positive than hospital nurses in various regards: relatively more home care nurses reported that they provided care in the physical and psychological domains, they were more positive about the quality of palliative care and they were more likely to feel competent to give this care. Considering the number of people who receive palliative care and die in hospital, it is important to keep educating and training nurses working in hospitals in providing palliative care. Even though the quality of palliative care was rated very good to excellent, improvements can still be made in both home care and hospitals. The palliative phase should be identified in good time and not just shortly before death. Timely identification is the first step in advance care planning, resulting in better quality of palliative care, in line with the individual patient’s needs and preferences.

## Data Availability

The datasets used and/or analysed during the current study available from the corresponding author on reasonable request.
